# A mini-review: recent advancements in temporal interference stimulation in modulating brain function and behavior

**DOI:** 10.3389/fnhum.2023.1266753

**Published:** 2023-09-14

**Authors:** Zhiqiang Zhu, Lijun Yin

**Affiliations:** School of Sport, Shenzhen University, Shenzhen, China

**Keywords:** temporal interference, transcranial electric stimulation, behavior, brain stimulation, brain function

## Abstract

Numerous studies have assessed the effect of Temporal Interference (TI) on human performance. However, a comprehensive literature review has not yet been conducted. Therefore, this review aimed to search PubMed and Web of Science databases for TI-related literature and analyze the findings. We analyzed studies involving preclinical, human, and computer simulations, and then discussed the mechanism and safety of TI. Finally, we identified the gaps and outlined potential future directions. We believe that TI is a promising technology for the treatment of neurological movement disorders, due to its superior focality, steerability, and tolerability compared to traditional electrical stimulation. However, human experiments have yielded fewer and inconsistent results, thus animal and simulation experiments are still required to perfect stimulation protocols for human trials.

## 1. Introduction

Neurological disorders are serious health challenges associated with clinical neurological and psychiatric conditions that cause a functional deterioration in motor, sensory, and behavioral functioning (Phillips, 2019; Gilmour et al., 2020; Lee et al., 2022a). Individuals with such disorders, such as Parkinson’s disease, frequently face impediments that interfere with their daily lives which burdens the healthcare system and society considerably (Grossman et al., 2018; Alonto et al., 2022). Consequently, efficient approaches are necessary to reduce the severity of neurological behavioral disorders.

Pharmaceutical strategies have been employed to combat neurological diseases and reduce their symptoms (Rossetto et al., 2021; Vardaxi et al., 2022). Nevertheless, this approach can affect neural activity across the entire brain, potentially resulting in undesired results (Vatansever et al., 2021). This limitation of pharmaceutical strategies has prompted the development of novel approaches. Non-pharmaceutical strategies such as transcranial electrical and magnetic stimulations stimulate specific brain regions, resulting in effective clinical treatments (Lozano, 2017; Grossman et al., 2018). These approaches have been used to treat depression and Parkinson’s disease. However, these therapies cannot achieve focal stimulation of the deep brain structures (Lozano, 2017).

To counter the inadequacies of current transcranial stimulation approaches, Grossman et al. created a novel non-invasive brain stimulation method called Temporal Interference Stimulation (TI). TI can produce a low-frequency envelope in the deeper parts of the brain, and modulate the activation of neurons. It eliminates the hindrance of deep invasive brain stimulation, necessitating the implantation of electrodes in the brain (Grossman et al., 2017). Studies have explored the influence of TI stimulation on brain function and behavior from various perspectives, including animal, human, and computer simulation studies. However, no study has summarized the studies about TI. Therefore, this review has summarized the existing research on TI, providing a foundation for further study and application of TI in humans.

## 2. Temporal interference stimulation

**Figure 1:**
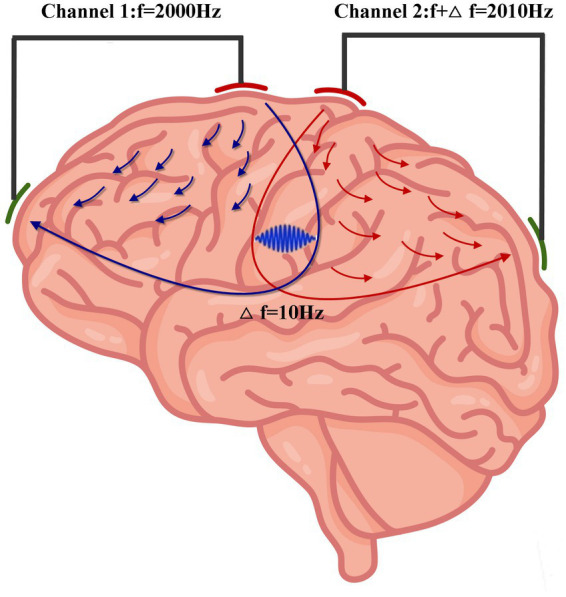
Concept of TI stimulation. Two high-frequency transcranial alternating stimulations with a slight frequency difference of 10 Hz (Channel 1: *f* = 2,000 Hz; Channel 2: f2 = 2,010 Hz) produce an envelope with a frequency of 10 Hz in the intersecting regions. (Some elements of this figure are from Vecteezy, https://www.vecteezy.com/).

TI involves the application of two high-frequency transcranial alternating currents of slightly different frequencies. The interaction of the two alternating currents produces a low-frequency envelope (Grossman et al., 2017). As the neural membrane is responsive to low-frequency electrical signals but not high-frequency signals (Hutcheon and Yarom, 2000), TI can focally stimulate the deep brain tissues without impacting the overlying and surrounding brain tissues (Figure 1). Moreover, the target area can be modulated by changing the current amplitude ratio of two pairs of electrodes (Grossman et al., 2017).

## 3. Preclinical studies

### 3.1. In vitro

Grossman et al. (2017) initiated a study using the mouse brain to examine the effectiveness of TI. In this experiment, 10 Hz TI (Channel 1: 2000 Hz; Channel 2: 2010 Hz) successfully activated c-fos expression in the mouse hippocampus without affecting the overlaying cortex. Similar to the study of Grossman, Carmona-Barrón et al. (2023) revealed that TI preserved a higher stimulation efficiency in the directed area of the deep brain structure and enhanced the immunoreactivity of rat blood–brain barrier cells.

### 3.2. Animal models

Grossman further demonstrated that TI stimulation induced the mouse’s forepaw and whisker movements, and the frequency of movement was in accordance with the frequency of the envelope (Grossman et al., 2017). Subsequently, Song et al. (2021) synchronously monitored the Ca + signals in the deep layers of the mice’s superior colliculus and the eye movements. They found that by altering the frequency of TI stimulation, it was possible to control the neural activity of the mice’s superior colliculus and regulate their eye movements.

In summary, these animal studies demonstrated that TI can effectively regulate brain function and behavior in rats. Compared to conventional tES, TI offers more precise stimulation of deep brain tissue and the capacity to alter the target area without moving the electrodes. However, there are limitations to consider. Specifically, the stimulation current intensity was more restricted in humans than in animal experiments.

## 4. Human studies

Five studies were conducted to evaluate the effects of TI on human behavioral or neurophysiological function. In neurophysiological investigation, Acerbo et al. (2022) used stereoelectroencephalography electrodes to compare electric field distributions in the brains of two cadavers during TI stimulation and tACS. This study measured brain discharge by implanting 12 stereoelectroencephalography electrodes in the temporal lobe on both sides and concluded that 130 Hz TI better penetrated the human hippocampus than the 130 Hz tACS. Zhu et al. (2022) investigated the effects of TI stimulation on the functional connectivity of the global brain. The study recruited 40 healthy young individuals and collected resting-state functional magnetic resonance imaging data before and during 20 Hz TI stimulation. The results showed that targeting the primary motor cortex (M1) with 20 Hz TI would enhance the functional connectivity between the M1 and the secondary motor cortices. However, the study of von Conta et al., 2022 did not find a change in EEG activity during a simple visual change-detection task before and after TI stimulation. Compared with sham stimulation, TI stimulation did not affect alpha-band brain oscillations. This could be due to the low stimulation intensity, which was only increased to 1 mA in this study. TI stimulation is characterized by a kilohertz frequency likely to lose some current intensity when penetrating the brain (Kondgen et al., 2008). With lower electrical stimulation intensities, it becomes more challenging to detect stimulation effects.

In the behavioral investigation, Zhang et al. (2022) randomly assigned participants to active-TI, sham-TI, active-transcranial alternating current stimulation (tACS), and sham tACS groups and measured their accuracy, reaction time (RT), and inverse efficiency scores on the N-back working memory tasks before, during, and after stimulation. The results indicated that the TI group exhibited enhanced working memory in high-load cognitive tasks than the sham-tACS group. Apart from our team, other research groups have examined the impact of TI on human performance. Moreover, Ma et al. (2021) studied its impact on a random reaction time task, a serial reaction time task (SRTT), and motor cortex excitability. The study revealed that 70 Hz TI stimulation benefited the RT in a random reaction time task and the excitability of the motor cortex. Additionally, 20 Hz TI stimulation substantially improved motor learning in the SRTT, which was linked to the increased motor-evoked potentials. These studies provide evidence that TI stimulation modulates brain function and improves human performance in terms of resting-state brain function, cognition, and behavioral performance. However, they do not provide data on the distribution of electric fields in the brain during TI stimulation.

Studies on humans have demonstrated that compared to tACS, TI has greater permeability in transcranial stimulation. Moreover, TI can effectively adjust functional brain networks, boosting cognitive function and neural excitability. However, the results of TI studies on human neurophysiology are inconsistent. Therefore, more studies on human neurophysiology and behavior are needed to provide solid evidence for the effect of TI.

## 5. Simulation studies

Simulation studies have focused on scrutinizing algorithms and perfecting TI strategies. Algorithms have been continuously optimized, significantly increasing the speed of simulations in TI stimulation. Bahn et al. (2023) developed an unsupervised neural network for simulating TI stimulation on the human brain that could rapidly and precisely optimize stimuli at deep brain targets. Moreover, Stoupis and Samaras (2022) discovered that using a genetic algorithm could drastically reduce the optimization time, making it possible to create personal stimulation plans rapidly. Research has indicated that unsupervised neural networks and genetic algorithms can be effective TI simulation algorithms. While both unsupervised learning and genetic algorithms can simulate TI-induced electric fields, no study has compared their relative efficiencies.

In optimizing stimulation montages, computer simulations offer numerous applicable scenarios for TI research to enhance intensity and focality in the targeted area. Regarding stimulation intensity, Lee et al. (2020) employed three realistic finite element human head models to modulate the position and current intensity of two pairs of electrodes. Delivering TI with an amplitude above the 0.2 V/m modulation threshold was possible by optimizing the TI electrode conditions. Rampersad et al. (2019) presented optimal four-electrode current patterns for the human brain and maximized the TI current to 0.37 V/m in the pallidum, 0.24 V/m in the hippocampus, and 0.57 V/m in the motor cortex. Concerning the stimulation focality, Lee et al. (2022c) employed finite element method-based electric field simulations of human brain models to demonstrate that epidural TI produced a more focal and stronger current in three targets (the anterior hippocampus, subthalamic nucleus, and ventral intermediate nucleus), and results were confirmed via phantom experiments. Huang and Datta (2021) and Lee et al. (2022b) simulated a multipair electrode strategy on the human head and observed that multipair TI electrodes offer a more focused stimulation than traditional TI using two-pair. These studies suggest that optimizing the electrode conditions and increasing the number of electrodes effectively improve TI efficiency.

In summary, computer simulations yield valuable information that can assist us in optimizing TI stimulation protocols.

## 6. The mechanism of temporal interference

Determining the mechanism of how TI influences brain function and behavior, allows for a more effective clinical application of TI. Currently, investigations of the mechanisms of TI effects on the brain have been conducted at the levels of animal models and computer simulations. Through their *in vitro* experiments and animal models, Grossman revealed the properties of TI stimulation to be focal and steerable. TI applied two kHz-frequency electric currents with a slight disparity to generate low-frequency electrical field envelopes. The envelopes improved the activation of the mice’s hippocampus without affecting its overlying cortex. Additionally, altering the current intensity ratio between the electrode pairs allows the maximum envelope at the deep site to be controlled without shifting the electrodes (Grossman et al., 2017).

By computer simulation approaches, Mirzakhalili et al. (2020) investigated the physics of TI neurostimulation from the perspective of ion-channel-mediated currents in three aspects to elucidate the mechanism more clearly. First, when a subthreshold TI stimulus was applied, the Na^+^ current was the most prominent among the ion currents passing through the axonal membrane, leading to a total inward current and causing the axons to depolarize. Upon reaching the axonal membrane potential threshold, an action potential was initiated, consistent with previous studies (Grossman et al., 2017). They suggested that the depolarizing property is linked to the resonance behavior of the axon, suggesting that the frequency of envelope coupling with the axon may improve TI efficiency. Second, they examined the initiation of action potentials along the axons when exposed to TI stimulation. Amplitude modulation of the electric field was confirmed as an essential element in initiating the action potential in the axon. The area of maximum amplitude modulation can be altered by modifying the current ratio among the electrode pairs, thereby allowing the stimulus target region to be modified. Third, the neural responses during TI can be classified into multiple categories. Grossman’s study determined that the neural response to TI stimulation is activated or inactive. In contrast, Mirzakhalili et al. (2020) illustrated multiple classes of neural responses during TI. The study revealed that the axon displayed physic activity when stimulated at the midline (*y* = 0.0 mm), tonic neural activity at *y* = 1.7 mm, and conduction block at 3.5 mm away from the midline.

Studies have examined factors affecting the effectiveness of TI. Plovie et al. (2022) employed a simplified Hodgkin-Huxley model of neural activity to assess the influence of TI with various parameters and proposed that the input stimulation intensity is critical in modulating the neuron membrane potential. Consistent with the study by Plovie, Gomez-Tames et al. (2021) employed a multiscale mouse brain model to explore the neural response to TI and verified that a greater current at a higher carrier frequency is needed to surpass the low-pass filter of the neuromembrane. Moreover, Von Conta et al. (2021) revealed that the electrical field strength at the target areas differed among individuals, suggesting that considering individual variability could enhance the effectiveness of TI. Wang et al. (2023) applied biophysically realistic neuron models and conducted simulations using various E-field stimulation parameters, assuming a uniform field. Subsequently, the total TI E-field vector rotated with the phase difference of the individual E-fields, implying that the three-dimensional characteristics of the TI E-fields should be considered in experimental investigations. Therefore, individual differences, three-dimensional spatial characteristics, and increasing the stimulation intensity at target areas, may be primary factors affecting the efficacy of TI.

## 7. Safety issue

Two studies have been conducted to examine the safety of TI stimulation from animal models and humans. At the animal level, Grossman et al. (2017) used immunohistochemistry to investigate the cellular and synaptic molecular profiles in the cortex and hippocampus of mice after 20 min of TI stimulation (2 kHz and 2.01 kHz). The profiles of the cellular and synaptic molecules remained unchanged. The temperature in the cortex beneath the lateral electrode increased slightly to 0.069 ± 0.05°C. At the human level, Piao et al. (2022) explored the safety of TI as regards influencing the human brain. The analyses revealed no considerable differences between the active (2 mA, 20/70 Hz, and 30 min) and sham (0 mA, 0 Hz, and 30 min) groups in various neurological and neuropsychological parameters, and no adverse effects were reported. Therefore, the protocols of current TI studies are safe.

## 8. Discussion

Studies have investigated the effects of TI on brain function and behavior. In comparison to traditional tES, TI has benefits in focality, steerability, and tolerance. The advantages of TI make it a promising treatment for neurological motor dysfunction diseases, such as Parkinson’s. Nevertheless, fewer studies have been done to assess the effects of TI on human neurophysiological function and behavior, and the results have been inconsistent. This situation may be attributable to a variety of causes. First, the current intensity delivered into the human brain is too low. It is insufficient to evoke a noteworthy neurophysiological and behavioral reaction in the human brain. Second, there is a wide variation of stimulation protocols used in existing TI studies, and the design of these protocols fails to consider the correlation between neural activity frequency and the frequency of stimulation, as well as the effect of functional brain networks on behavior. Third, the approaches currently utilized in TI studies of behavioral measurement are overly simplistic and limited in scope.

To improve upon the shortcomings of existing research on TI, future studies could take into account factors such as:

### 8.1. Securely increasing the modulation amplitude of electric currents in humans

The modulation amplitude of electric currents is critical to the effectiveness of TI. Following operational guidelines of conventional transcranial electric stimulation (tES), most TI studies have set the stimulation intensity at 0–2 mA (Zhang et al., 2022; Zhu et al., 2022). Nevertheless, the impedance of TI stimulation is notably lower than that of low-frequency tES, which allows for the application of higher stimulation intensity (Ariel et al., 2019; Rampazo and Liebano, 2022). Moreover, TI has a higher tolerance than conventional tES (Liu et al., 2021). Therefore, within safe operation parameters, increasing the intensity of TI can be taken into consideration.

### 8.2. Stimulating in closed-loop

The frequency coupling between TI envelopes and axonal behaviors affects the efficiency of TI stimulation (Mirzakhalili et al., 2020), thus necessitating the evaluation of neural activity frequency to determine the frequency of TI stimulation. Future studies could consider a closed-loop stimulation mode, which incorporates an electroencephalogram to detect neural activity or examine the frequency of limb movements that are coupled with neural activity, to obtain the stimulation protocol.

### 8.3. Stimulating a functional network with multiple targets

Human behavior is not governed by a single region of the brain, rather the collaboration of multiple brain regions is responsible for its control (Park and Friston, 2013; Marek and Dosenbach, 2018). Simultaneously stimulating multiple targets in the brain’s functional network may help regulate particular functions.

### 8.4. Temporally interfering transcranial magnetic stimulation

Temporally Interfering Transcranial Magnetic Stimulation (TI-TMS) is a novel approach that achieves Temporal Interference Stimulation in a targeted brain area via the Transcranial Magnetic Stimulation technique (Sorkhabi et al., 2020; Xin et al., 2021). Xin et al. (2021) and Sorkhabi et al. (2020) determined the efficiency of TI using computer simulations, which demonstrated that TMS with temporal interference could activate neural activity without influencing the cortex above it and that the stimulation targets could be directed without adjusting the coil positions. Compared with conventional TMS, temporal interference TMS can provide a deeper and more focal brain stimulation. Based on this, Khalifa et al. (2023) developed a novel two-solenoid coil to stimulate temporal interference. The results of the experiments on mice revealed that temporal interference with TMS could significantly increase the expression of c-Fos in the deeper layers of the mouse brain without affecting the overlaying tissue. Therefore, TI-TMS exhibits excellent potential as a tool for managing human behavior.

### 8.5. Optimizing the behavioral tests

Tests currently employed to validate the behavioral effects of TI are open to bias, thus it is suggested that more dependable behavioral tests be utilized to bolster the reliability of behavioral data. For example, using Short Latency Afferent Inhibition (SAI) as a neurophysiological marker (Bonnì et al., 2017), and collecting the SAI data in different phases during TI stimulation to better understand the effect of TI on central cholinergic activity.

## 9. Conclusion

TI is a promising technology for the treatment of Neurological Movement Disorders, due to its superior focality, steerability, and tolerability compared to traditional electrical stimulation. However, human experiments have yielded fewer and inconsistent results, thus animal and simulation experiments are still required to perfect stimulation protocols for human trials.

## Author contributions

ZZ: Conceptualization, Funding acquisition, Investigation, Methodology, Writing – original draft. LY: Conceptualization, Funding acquisition, Project administration, Supervision, Writing – review & editing.

## Funding

The author(s) declare financial support was received for the research, authorship, and/or publication of this article. This work was supported by the National Natural Science Foundation of China (11932013); The Educative Reform Project of Shenzhen University (JG2022015); The Shenzhen Stability Support Program (20220810110849002).

## Conflict of interest

The authors declare that the research was conducted in the absence of any commercial or financial relationships that could be construed as a potential conflict of interest.

## Publisher’s note

All claims expressed in this article are solely those of the authors and do not necessarily represent those of their affiliated organizations, or those of the publisher, the editors and the reviewers. Any product that may be evaluated in this article, or claim that may be made by its manufacturer, is not guaranteed or endorsed by the publisher.

## Abbreviations

TIS: temporal interference stimulation
RT: reaction time
SRTT: serial reaction time task
tACS: transcranial alternating current stimulation
tES: transcranial electric stimulation
TMS: transcranial magnetic stimulation
